# Outcome of primary deltoid ligament repair in acute ankle fractures: a meta-analysis of comparative studies

**DOI:** 10.1007/s00264-019-04416-9

**Published:** 2019-11-27

**Authors:** Motasem Salameh, Abduljabbar Alhammoud, Nedal Alkhatib, Ahmed K. Attia, Mohamed M. Mekhaimar, Pieter D’Hooghe, Karim Mahmoud

**Affiliations:** 1 Orthopedic Surgery Department, Hamad General Hospital, PO Box 3050, Doha, Qatar; 2 Aspetar Orthopedic and Sports Medicine Hospital, Doha, Qatar; 3 University of Pennsylvania Foot and Ankle Program, Philadelphia, PA USA

**Keywords:** Ankle, Deltoid, Fracture, Instability, Ligament

## Abstract

**Purpose:**

The indications of deltoid ligament repair in ankle injuries with widened medial clear space in the absence of medial malleolus fracture remain controversial. Many authors reported no difference in long-term functional outcomes, while others stated that persistent medial clear space widening and malreduction are higher when deltoid ligaments went without repair. This meta-analysis aims to report the current published evidence about the outcomes of deltoid ligament repair in ankle fractures.

**Methods:**

Several databases were searched through May 2018 for comparative studies. The primary outcome was the medial clear space correction, while secondary outcomes included maintenance of medial clear space reduction, pain scores, functional outcome, and total complications if any. Three comparative studies met the inclusion criteria for the meta-analysis. The analysis included a total of 192 patients, 81 in the deltoid ligament repair group and 111 in the non-repair group.

**Results:**

The medial clear space correction and maintenance of the said correction on final follow-up radiographs were superior in the deltoid ligament repair group. Although the pain scores were better in the repair group at the final follow-up, this did not result in a better functional outcome, with similar total complication rates.

**Conclusion:**

In conclusion, those who had their deltoid ligament repaired had superior early and late radiological correction of the medial clear space, an indicator of the quality of ankle reduction with better pain scores. However, no differences in the functional outcome and complications rate were reported.

## Introduction

The deltoid ligament (DL) is considered to be the main stabilizer of the ankle joint; it consists of both superficial and deep parts and extends from the medial malleolus to the talus, calcaneus, and navicular bones [1, 2]. The superficial deltoid is the primary restraint to hind foot eversion, while the deep deltoid is the primary restraint to ankle external rotation [3]. The DL can be injured from supination external rotation (SER), pronation external rotation (PER), and pronation-abduction ankle fractures [4, 5]. The superficial and deep deltoid must be completely ruptured to render the ankle unstable, with abnormal talus motion [6–8].

DL rupture with ankle fracture is not uncommon and can be underdiagnosed and undertreated [9]. Isolated lateral malleolus fracture with medial side pain, bruising, ecchymosis, or opening of the medial clear space (MCS) can indicate injury to the medial components of the ankle joint “bimalleolar equivalent.” A wide MCS is defined as more than 4 mm on a nonstressed mortise view and at least 1 mm greater than the superior tibiotalar clear space [10]. The MCS equal or greater than 5 mm on stress radiographs is considered to be a diagnostic key for DL rupture [8, 11].

Several techniques have been proposed for the treatment of DL rupture, such as simple primary repair, anchor sutures, or graft reconstruction. The outcome of DL repair remains controversial, whereas older literature showed no significant difference after DL repair, while recent studies showed superior outcomes.

This meta-analysis aims to report the outcome of DL repair in ankle fractures in terms of maintenance of MCS reduction, functional outcomes, and complication rates.

## Materials and methods

This article was performed following the Preferred Reporting Items for Systematic Reviews and Meta-Analyses (PRISMA) guidelines [12].

### Literature search

Relevant comparative studies in the literature were identified from database inceptions through May 2018. An electronic-based search on MEDLINE (PubMed), EMBASE, Google Scholar, and Cochrane database was performed using the following keywords and their synonyms: (“ankle” AND “fracture,” AND “deltoid”). The reference lists from previous meta-analyses and review articles were searched manually for eligible studies.

Two investigators independently reviewed all titles, abstracts, and the full text of potentially related articles, based on abstract reviews. Studies were selected by inclusion and exclusion criteria, with any disparity resolved by the senior author.

### Study eligibility criteria

The research team reviewed published studies as per the following inclusion criteria: comparative studies on the effect of DL repair in acute ankle fractures, reporting one of the following desirable outcomes: MCS measurement on preoperative, postoperative, and final follow-up x-rays, functional outcomes, pain scores, and complication rates with a minimum follow-up of 12 months. Studies not reporting any of the outcomes of interest or the full text is not available in English were excluded.

The primary outcome was the maintenance of the MCS at final follow-up, whereas secondary outcomes were (1) MCS correction, (2) functional outcome using the American Orthopaedic Foot and Ankle Society (AOFAS) score, (3) pain scores using the visual analogue scale (VAS), and (4) complication rates.

### Risk of bias assessment

The Newcastle–Ottawa Quality Assessment scale [13] was used for quality and bias assessment by two independent investigators. The Newcastle–Ottawa scale examined study quality for three points (selection, comparability, and outcome).

### Data collection

The data retrieved included study characteristics (name, year, level of evidence, and follow-up period), subjects’ characteristics (sample size and age), management characteristics, and outcome measures.

### Data analysis

Data analysis used comprehensive meta-analysis software, with a random-effect model and SPSS 22 (IBM, Armonk, NY, USA). For continuous variables, standardized mean difference (SDM) and 95% confidence interval (CI) were calculated. Level of evidence was based on the Cochrane Book Review Group [14].

## Results

**Fig. 1:**
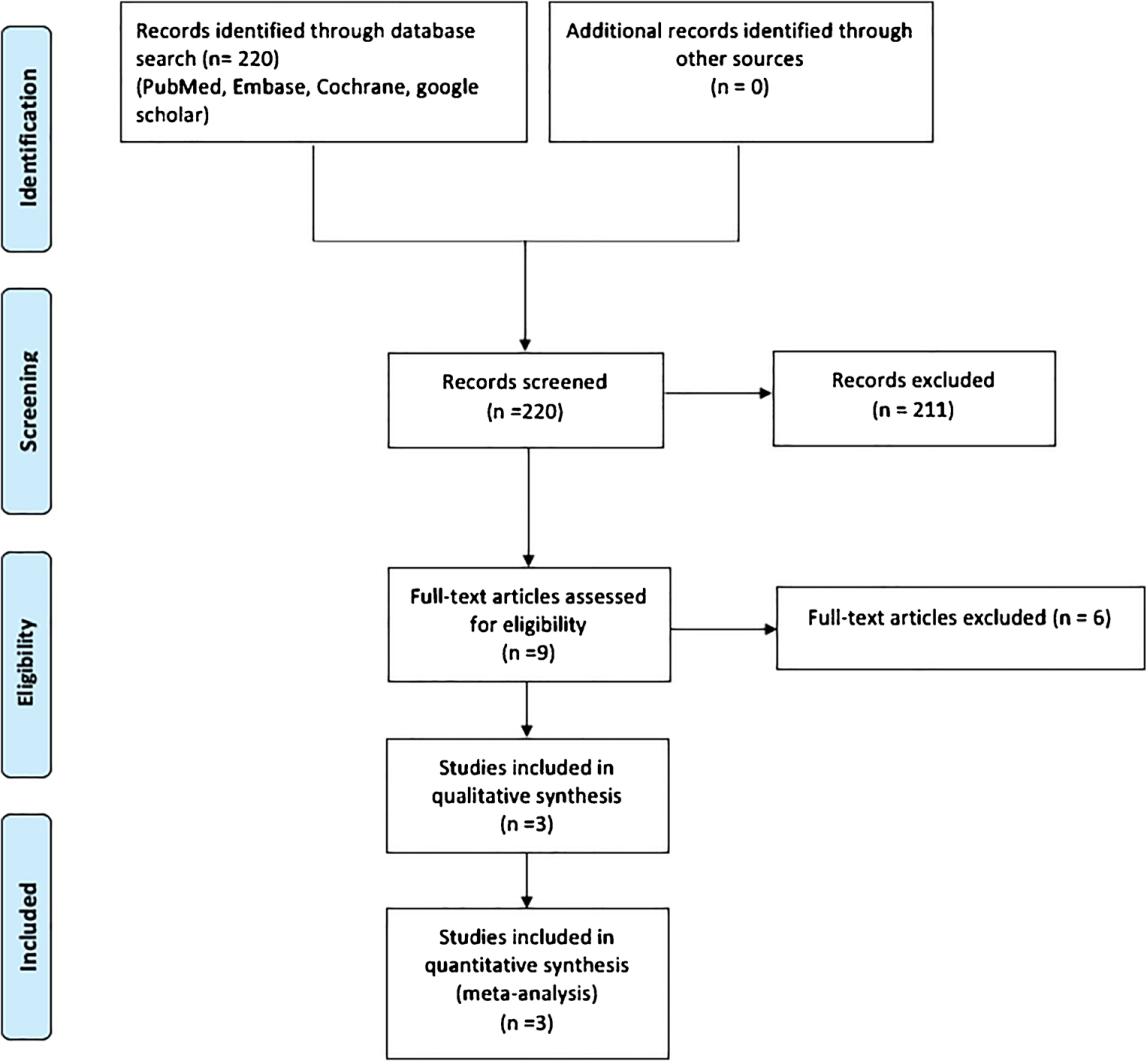
PRISMA chart

After exclusion of duplicates and non-English articles, there were 220 titles and abstracts reviewed, of which nine full-text studies were eligible. Of these studies, only three met inclusion and exclusion criteria, while the other six were excluded for not reporting any of the outcome measures of interest (Fig. 1).

### Demographics

Three comparative studies were eligible for meta-analysis, for a total of 192 patients with ankle injuries, 81 in the DL repair group and 111 in the non-repair group. The mean age was 40.5 years in the repair group and 37.8 years in the non-repair group. Minimum follow-up of all subjects was 12 months (Table 1).

**Table 1:** Summary of included studies

Study/year	Country	Design	No. of patients analyzed	Age average in years	Outcome measures analyzed	Follow-up period (minimum)
			Total ( repaired, unrepaired)			
Gu et al. [15] 2017	China	Prospective Level II	40 (20/20)	Treatment: 40.6 Control: 37.5	AOFAS score, MCS, VAS, complications	12 months
Woo et al. [16] 2017	Korea	Retrospective Level III	78 (41/37)	Treatment: 41.6 Control: 39.4	AOFAS score, MCS, VAS, complications	12 months
Zhao et al. [17] 2017	China	Retrospective Level III	74 (20/54)	Total: 39.5	AOFAS score, MCS, VAS, complications	14 months

*AOFAS*, American Orthopedic Foot and Ankle Society; *MCS*, medial clear space; *VAS*, visual analogue scale

### Quality assessment

Table 2 summarizes the results of the different domains of study quality, as adapted from the Newcastle–Ottawa Scale [10]. All studies were judged on eight items and categorized into three groups: selection of study groups, comparability of groups, and assessment of the outcome of interest. A total of nine stars deemed the study to be of the highest quality.

**Table 2:** Newcastle–Ottawa Quality Assessment of the included studies in the meta-analysis

Domain	Item	Gu et al.	Woo et al.	Zaho et al.
Selection (maximum of 4 stars)	Representativeness of the exposed cohort	*	*	*
	Selection of the non-exposed cohort	*	*	*
	Ascertainment of exposure	*	*	*
	Demonstration that outcome of interest was not present at start of study	*	*	*
Comparability (maximum of 2 stars)	Comparability of cohorts on the basis of the design or analysis	*	**	**
Outcomes (maximum of 3 stars)	Assessment of outcome	*	*	*
	Was follow-up long enough for outcomes to occur	*	*	*
	Adequacy of follow-up of cohorts	*	*	*

### Results of individual studies

#### Indication for deltoid repair

Gu et al. [15] included patients with MCS ≥ 5 mm on preoperative stress radiographs, with magnetic resonance imaging (MRI) confirming both superficial and deep deltoid. Woo et al. [16] repaired the DL in patients with MCS > 4 mm, MCS 1 mm greater than the superior tibiotalar clear space, or any lateral tibial shift on intraoperative stress views after fixation of the lateral malleolus and disrupted syndesmosis if observed. Zhao et al. [17] included adult patients with MCS ≥ 6 mm on preoperative anteroposterior ankle radiographs.

#### Surgical technique

Ankle fracture dislocations with isolated lateral malleolus fractures were included in the study of Gu et al. [15]; after fixation of the lateral malleolus, a medial incision was made with repair of the DL using anchor sutures. Woo et al. [16] included patients with SER or PER ankle injuries, with an isolated lateral malleolus fracture, after repairing the lateral malleolus plus/minus syndesmotic fixation, as indicated by the cotton test [18]; a medial incision was made and the DL was fixed with 1 or 2 anchor sutures in the medial malleolus, 5 mm lateral to the medial talus. Zhao et al. [17] repaired the DL in SER, PER, and pronation-abduction injuries, after fixation of the lateral malleolus fractures, indicated posterior malleolus fractures and disrupted syndesmosis, as observed by intraoperative images. The DL was repaired through a medial incision, direct suturing to the talus or medial malleolus augmented with anchor suture, and suturing of the superficial deltoid with an absorbable material.

#### Medial clear space

Zhao et al. [17] reported a significantly small MCS in the DL repair group postoperatively and at final follow-up (*P* = 0.03). Woo et al. [16] reported a significant difference in the average final follow-up for MCS between the two groups (*p* = 0.001), but there was no difference seen in the immediate postoperative average MCS. Gu et al. [15] found a significant reduction in the MCS between preoperative and postoperative radiographs in both groups at 1 year follow-up, with a greater improvement in the DL repair group (*P* = 0.02).

#### Functional outcome

The AOFAS score was reported in all included studies. Gu et al. [15] reported significantly better AOFAS scores after DL repair (*P* = 0.001); yet, in the two studies by Woo et al. [16] and Zhao et al. [17], the AOFAS scores were comparable in the two groups, with no statistical significance (*P* > 0.05). Woo et al. [16] found no difference in foot function index (FFI) between the two groups.

Woo et al. [16] further compared the functional outcomes in fractures that underwent syndesmotic fixation, and reported a significantly better AOFAS and FFI in the DL repair group (*P* = 0.02).

#### Pain score

VAS pain score was reported by three authors; although no significant difference reported between the two groups by Woo et al. [16] and Zhao et al. [17], Gu et al. [15] reported a significantly lower VAS score after DL repair (*P* = 0.02).

#### Complications

Gu et al. [19] reported no statistical significance (*P* > 0.05) in the incidence of complications between the two groups, despite longer surgical time (*P* = 0.026) and greater blood loss (*P* = 0.032) in the DL repair group. Woo et al. [16] similarly reported a significantly longer operative time in the DL group (*P* < 0.01) with no intraoperative or final follow-up complications. A malreduction rate of 20.4% was reported by Zhao et al. [17] in the non-repair group, with 4 patients requiring revision surgery, but none requiring revision surgery in the DL repair group (*P* = 0.03).

### Meta-analysis

**Fig. 2:**
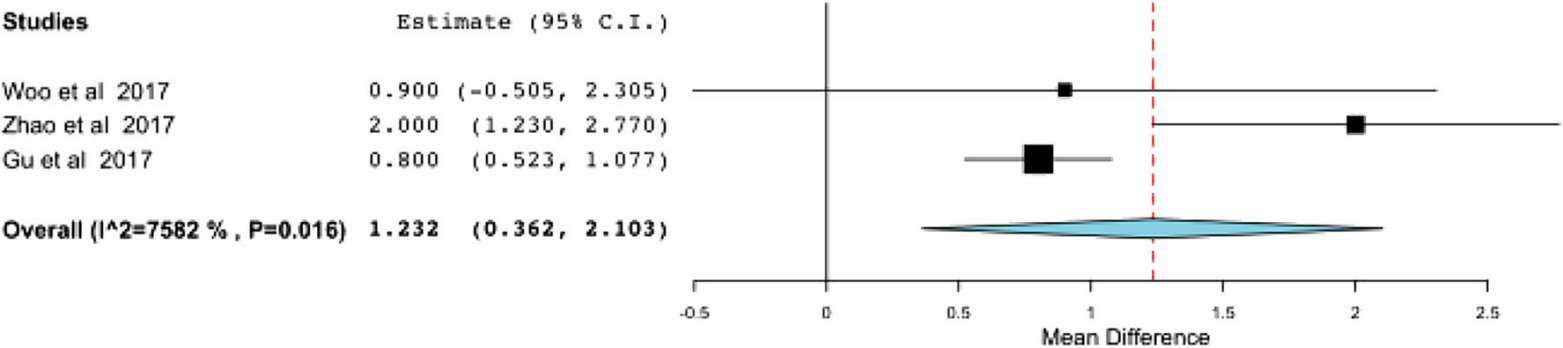
The effect of deltoid ligament repair on postoperative correction of the medical clear space. CI confidence interval

**Fig. 3:**
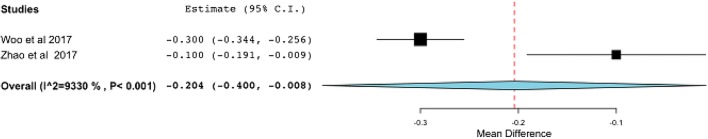
The effect of deltoid ligament repair on maintenance of medical clear space correction at final follow-up. CI confidence interval

The MCS correction was superior in the DL repair group with statistical significance (SDM, 1.232; 95% CI. [0.362, 2.103]) (Fig. 2); maintenance of the MCS correction at the final follow-up was also significantly better in the repair group (SDM, − 0.204; 95% CI, [− 0.400, − 0.008]) (Fig. 3).

**Fig. 4:**
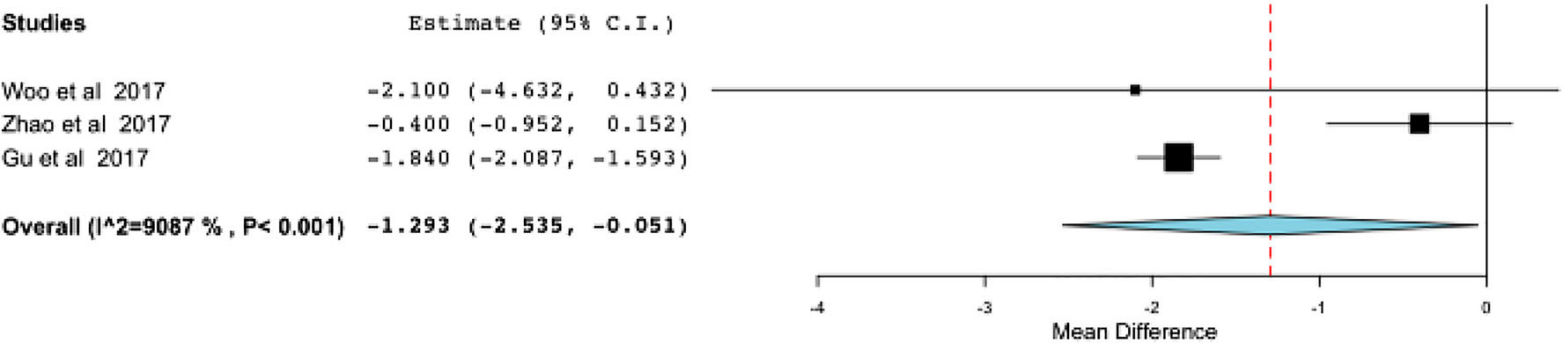
The effect of deltoid ligament repair on pain scores at final follow-up. CI confidence interval

**Fig. 5:**
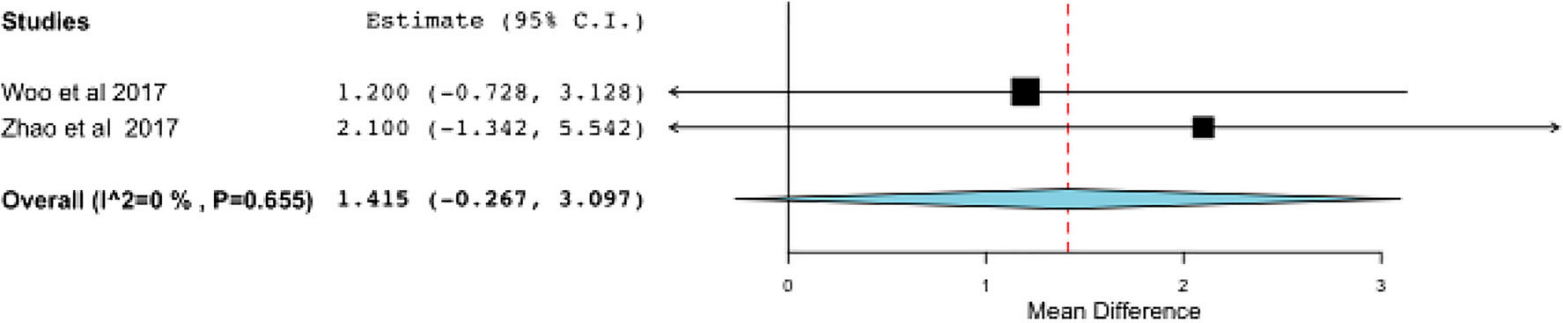
The effect of deltoid ligament repair on functional outcome at final follow-up. CI confidence interval

**Fig. 6:**
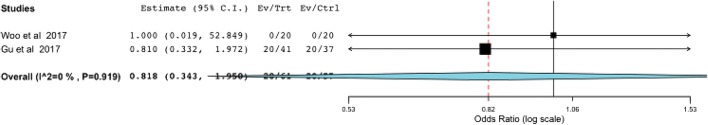
The effect of deltoid ligament repair on total complications rate. CI confidence interval

Although the VAS was lower in the repair group at final follow-up (SDM, − 1.293; 95% CI, [−2.535, − 1.051]) (Fig. 4), the functional outcome of AOFAS did not show any difference between the two groups (SDM, 1.415; 95% CI, [− 0.267, 3.097]) (Fig. 5), with equal total complication rates (OR, 0.818; 95% CI, [0.343, 1.950]) (Fig. 6).

## Discussion

In this meta-analysis on ankle fractures with widened MCS, DL repair was associated with superior and long-term reduction of the ankle, evident by the immediate postoperative difference in the MCS, as well as maintenance of the MCS at final follow-up of at least 12 months. Ankle mortise malreduction can lead to chronic ankle instability and ankle pain, and subsequent post-traumatic arthritis [20–22]. Ramsey et al. [20] reported a 49% increase in ankle joint pressure, with as little as 1 mm talar shift. Furthermore, Horisberger et al. [22], in their multicenter study, argued that postoperative ankle malalignment is considered to be a prognostic factor for developing post-traumatic arthritis. This was consistent with our results of lower pain scores in patients who had DL repair, as observed at the final follow-up.

General principles of ankle fracture management are restoring anatomical alignment and joint congruity, ensuring stability, and reducing long-term complications. Medial column (medial malleolus and DL) was reported to be more crucial to the stability of the ankle than the lateral component: DL acts as an effective medial restraint to stabilize the talus and guide normal physiological range of motion [6, 8]. Michelson et al. [8] in a cadaveric study found that a fibular osteotomy with superficial deltoid transection did not change the MCS on mortise views; however, combining superficial and deep deltoid transection resulted in 100% talar shift and valgus tilt. However, there is still no consensus among foot and ankle surgeons about repair of the DL in ankle fractures; earlier studies suggested that its repair was not necessary, with restoration of lateral malleolus integrity being sufficient to stabilize the ankle [23–25]. Stromsoe et al. [25] randomized 50 cases of Weber B and Weber C fractures with DL injury to study the effect of DL repair; they concluded that the DL can be left unrepaired with no effect on return to work, sports activities, or clinical symptoms at 17-month follow-up, on average. However, some recent and well-done studies suggested a better outcome after DL repair [15–17, 26, 27]. Hsu et al. [27], in his series of 14 National Football League (NFL) players with ankle fractures, found that open DL repair allowed return to play with no complications at 12-month follow-up. In another study by Yu et al [26], DL repair in 106 patients with acute ankle fractures resulted in satisfactory functional outcomes, with no clinical or radiological evidence of post-traumatic ankle arthritis in fractures managed with DL repair, as observed at an average of 27-month follow-up.

Whether repair of the DL affects functional outcome is still controversial; Gu et al. [15] in the only prospective comparative study with functional outcomes, reported 90% excellent and good AOFAS scores in the DL repair group compared with 60% in the non-repair group (*p* = 0.001). Furthermore, when comparing cases with syndesmotic fixation, an average AOFAS scores of 93.1 in the repair group and 89.8 in the non-repair group (*P* = 0.02) were reported by Woo et al. [16].

Some might argue that adding an extra surgical incision on the medial side would increase operative time and the risk of wound complications, but our data analysis showed no statistically significant difference in total complication rate. Woo et al. [16] and Gu et al. [15] reported longer operative time in the DL repair group, with an average of 17 and 70 minutes, respectively. Longer operative time should be weighed against the risk of chronic ankle pain and post-traumatic arthritis.

Limitations of this review are similar to all other meta-analyses, including heterogeneity of included studies, unknown bias in the primary studies, and the inclusion of articles published only in English. This can be reflected by design of the studies, in which we could not pool the data on the AOFAS scores, as found by Gu et al. [15]—as a function of being reported in a categorical manner rather than reporting a numerical score, which had been done by Woo et al. [16] and Zhao et al. [17]. This acknowledges that pooling data from two studies may be insufficient. We also could not pool data on the effect of DL repair on specific fracture types. Another limitation was the small number of studies included, as a result of our search identified only three studies in the literature to directly examine surgical repair of the DL in ankle fractures, compared with ankles treated with no repair of the ligament with a total of 192 patients.

To the best of our knowledge, this is the first meta-analysis to pool data from comparative studies regarding the effect of DL repair in ankle fractures with widened MCS; this information can be used in future randomized clinical trials on this topic.

DL injury in the context of ankle fracture is underdiagnosed and undertreated, yielding questions about proper management. DL repair in ankle fractures with widened MCS showed better anatomical reduction of the ankle, lower pain scores at final follow-up, and no significant increase in complication rate.
